# Multi-parameter ultrasonography-based predictive model for breast cancer diagnosis

**DOI:** 10.3389/fonc.2022.1027784

**Published:** 2022-11-17

**Authors:** Jing Chen, Ji Ma, Chunxiao Li, Sihui Shao, Yijin Su, Rong Wu, Minghua Yao

**Affiliations:** Department of Ultrasound, Shanghai General Hospital, Shanghai Jiao Tong University School of Medicine, Shanghai, China

**Keywords:** multi-parameter ultrasonography, diagnostic model, shear wave elastography, contrast-enhanced ultrasound, breast cancer

## Abstract

**Objectives:**

To develop, validate, and evaluate a predictive model for breast cancer diagnosis using conventional ultrasonography (US), shear wave elastography (SWE), and contrast-enhanced US (CEUS).

**Materials and methods:**

This retrospective study included 674 patients with 674 breast lesions. The data, a main and an independent datasets, were divided into three cohorts. Cohort 1 (80% of the main dataset; n = 448) was analyzed by logistic regression analysis to identify risk factors and establish the predictive model. The area under the receiver operating characteristic curve (AUC) was analyzed in Cohort 2 (20% of the main dataset; n = 119) to validate and in Cohort 3 (the independent dataset; n = 107) to evaluate the predictive model.

**Results:**

Multivariable regression analysis revealed nine independent breast cancer risk factors, including age > 40 years; ill-defined margin, heterogeneity, rich blood flow, and abnormal axillary lymph nodes on US; enhanced area enlargement, contrast agent retention, and irregular shape on CEUS; mean SWE higher than the cutoff value (P < 0.05 for all). The diagnostic performance of the model was good, with AUC values of 0.847, 0.857, and 0.774 for Cohorts 1, 2, and 3, respectively. The model increased the diagnostic specificity (from 31% to 81.3% and 7.3% to 73.1% in cohorts 2 and 3, respectively) without a significant loss in sensitivity (from 100.0% to 90.1% and 100.0% to 81.8% in cohorts 2 and 3, respectively).

**Conclusion:**

The multi-parameter US-based model showed good performance in breast cancer diagnosis, improving specificity without a significant loss in sensitivity. Using the model could reduce unnecessary biopsies and guide clinical diagnosis and treatment.

## Introduction

According to Global Cancer Statistics 2020, breast cancer has surpassed lung cancer as the leading cause of global cancer incidence, and it ranked first for incidence and mortality in the vast majority of countries among women (1). Compared with the traditional management of breast cancer, the current therapy shifts from surgical approach to precise and individualized treatment. Especially in recent years, with the development of molecular biology, a number of new biological markers for the prognosis of breast cancer, represented by WDR34 mRNA, provide novel target for the diagnosis and treatment of breast cancer (2). However, the satisfactory treatment effect depends not only on the change of treatment methods and prognosis judgment, but also on accurate preoperative diagnosis.

Ultrasound (US) is the most used modality for breast cancer detection and diagnosis among Chinese women, whose breasts are usually more denser compared to Caucasian women (3). With the advantages of convenient, non-ionizing, non-invasive, inexpensive, and provides real-time imaging, conventional US can provide useful information about breast lesions and the surrounding tissue (4). Unfortunately, although US has relatively high sensitivity, its moderate specificity, due to the small lesion size or atypical features, often leads to false positive findings and many unnecessary biopsies. Therefore, new US technologies were developed to supplement the conventional US, including shear wave elastography (SWE) and contrast-enhanced US (CEUS) (5, 6).

SWE can be used to estimate the stiffness of lesion qualitatively or quantitatively. The use of SWE, especially in combination with conventional US, has increased the diagnostic accuracy, compared to single mode US (7, 8). The stiffness can be assessed qualitatively by analyzing a color-scaled image and/or quantitatively by determining the mean and maximum elasticity values (kPa) as well as the ratio of maximum elasticity to adipose tissue. In this way, the color closer to red and the higher elasticity value or ratio indicate malignant lesions.

CEUS has been used in clinical practice to provide more information regarding tumor blood supply to differentiate benign from malignant breast lesions (9, 10). Abnormal blood perfusion or blood vessel filing patterns observed on CEUS images and videos could reveal perfusion characteristics associated with malignant tumors. Studies on the role of CEUS in the past decade have shown that CEUS could increase the specificity of conventional US (11–13).

Compared to the single mode US with obtain limited diagnostic information, multi-parameter US is considered to provide more systematic and comprehensive information. The diagnostic performance of SWE or CEUS combined with conventional US had been reported, but these studies only employed two of the three US modes (14, 15). Only a few studies have combined all three modes, whereas it did not include all breast lesions categories of BI-RADS 3 to 5, nor did it include quantitative analysis of SWE (16–18). Therefore, we aimed to develop, validate, and evaluate a diagnostic predictive model for breast lesion diagnosis (BI-RADS 3 to 5) using multi-parameter US (conventional US, SWE, and CEUS), comparing it to diagnosis by conventional US alone. The purpose of this study is to assess the value of multi-parameter US in the diagnosis of breast cancer, to invest whether it can improve the diagnostic efficiency, and reduce unnecessary breast biopsies.

## Materials and methods

### Patients

**Figure 1:**
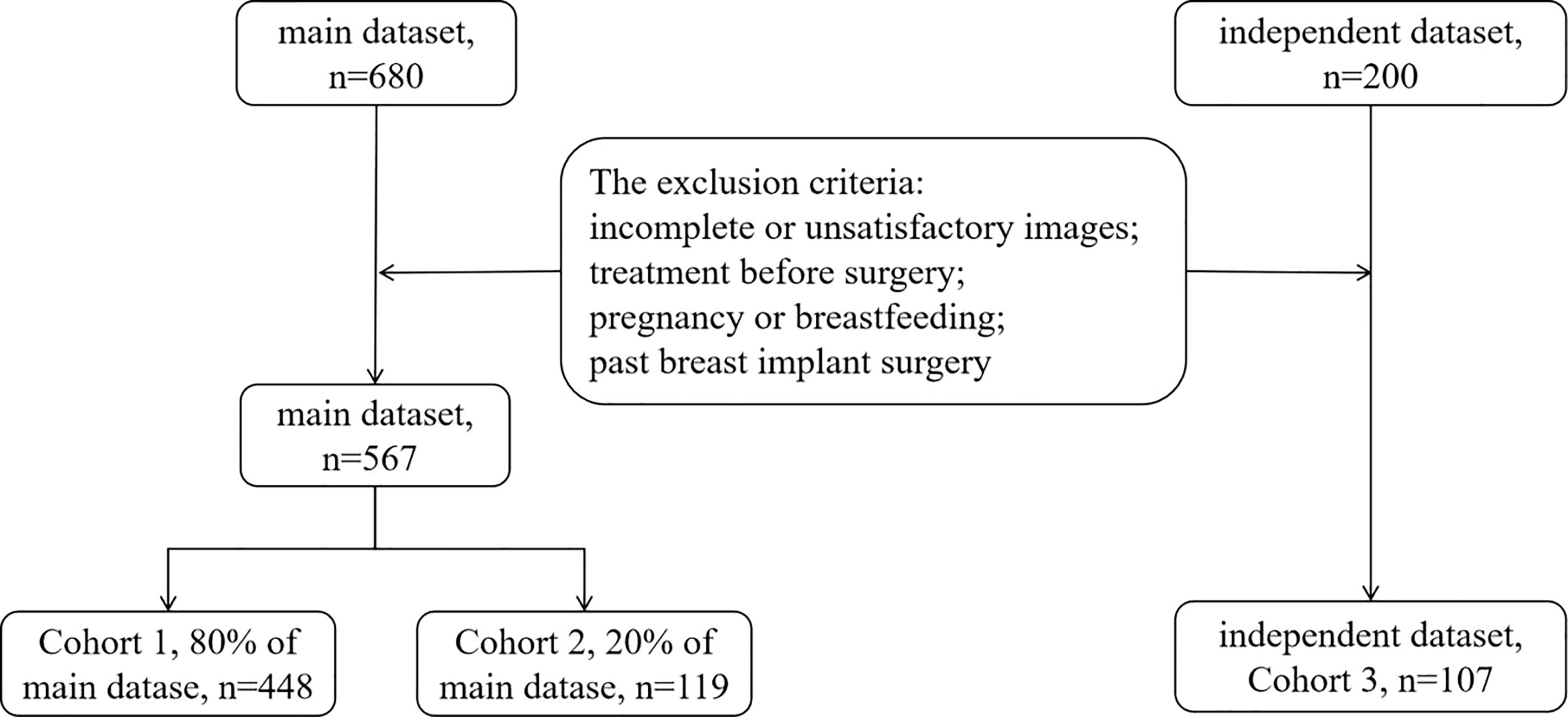
Patient enrollment flow chart.

This study retrospectively analyzed 674 consecutive patients (mean age, 47.26 ± 14.53; range, 18–94 years) with 674 pathologically-confirmed breast lesions treated at Shanghai General Hospital (Shanghai, China) from June 2018 to December 2020. The inclusion criteria were as follows: aged over 18 years; underwent conventional US, SWE, and CEUS examinations performed by the same sonographer with the same US machine, as was usually done when evaluating patients with a breast mass before surgery; available pathology results for each lesion after surgery or core needle biopsy. Thus, the study totally included 680 breast lesions in the main dataset and 200 breast lesions in the independent dataset.The exclusion criteria were incomplete or unsatisfactory images, treatment before surgery, pregnancy or breastfeeding, and past breast implant surgery. As a result, the main dataset finally included 567 breast lesions and the independent dataset finally included 107 breast lesions, as shown in **Figure 1**. The most suspicious or largest lesion was chosen in patients with multiple pathologically-confirmed lesions. This retrospective study was approved by the institutional ethics committee of Shanghai General Hospital, the patients signed informed consent forms before CEUS was performed, and all participating researchers were blinded.

The data were divided into three cohorts according to the two campuses of the hospital and in chronological order. The main dataset was divided into Cohorts 1 and 2. It included examinations conducted by two sonographers from the South Campus, both with over five years of experience in breast US. The independent dataset used for Cohort 3 included examinations conducted in the North Campus by a third sonographer with over five years of experience in breast US. Cohort 1 comprised 448 patients (80% of the main dataset; mean age, 46.10 ± 13.94; range, 18–94 years) assessed between June 2018 and November 2019. Their data were used to establish a predictive model for differentiating malignant from benign breast lesions. Cohort 2 comprised 119 patients (20% of the main dataset; mean age, 46.67 ± 14.25; range, 22–85 years) assessed between December 2019 and December 2020. This cohort was used to validate the developed predictive model. Cohort 3 comprised 107 patients (mean age, 52.77 ± 16.10; range, 22–87 years) assessed between June 2018 and December 2020. This cohort was used to evaluate the developed predictive model.

### Conventional US, SWE, and CEUS examination

All three US techniques were performed using the same US machine (APlio 500, TOSHIBA Medical Systems, Minato Ward, Tokyo, Japan) following the American Institute of Ultrasound Medicine guidelines. All patients were positioned with their breasts fully exposed. Conventional US was performed using a linear transducer (7–12 MHz), noting the lesion location, size, length-to-width ratio, margin (well-defined, ill-defined), shape (oval, irregular), internal echo (hypoechoic, isoechoic, hyperechoic), posterior echo (with or without attenuation), peripheral tissue distortion (with or without), microcalcification (with or without), blood flow (Adler grades II and III were defined as rich, Adler grades 0 and I were defined as non-rich), and axillary lymph node status (normal, abnormal). The images were stored.

Subsequently, SWE was performed using the same linear array transducer. A region of interest (ROI) that included the entire lesion and a small amount of surrounding tissue was drawn. The hand holding the probe was as light as possible to ensure accurate results. The differential diagnosis of benign from malignant breast lesions considered both qualitative and quantitative SWE aspects. The analysis was based on color, with red representing stiff and blue representing soft tissue. Five SWE images were recorded for the qualitative analysis.

Finally, CEUS was performed using a linear transducer (4–9 MHz). The target section selected for CEUS was based on the plane with the richest blood supply as visualized on conventional US. The most suspicious plane was selected if no plane with abundant blood supply was detected, e.g., the plane with the maximal diameter or one with an irregular shape. CEUS was performed in the dual-image mode to ensure accuracy of the results, and the mechanical index was set to 0.06. Sulfur hexafluoride microbubbles (4.8 mL; SonoVue^®^, Bracco Imaging S.p.A., Milan, Italy) were injected through the antecubital vein, followed by injection of 5–10 mL of saline. The videos and images were recorded for 180 s starting immediately after injection.

### Image analysis

Two skilled sonographers, different from the above and blinded to the pathological results and each other’s findings, analyzed all US images. Both had over five years of experience in conventional US, SWE, and CEUS for breast cancer diagnosis. Disagreements were resolved by discussion to reach a consensus. Lesions in the conventional US and CEUS images were classified following the Breast Imaging Reporting and Data System (BI-RADS) guidelines into categories 0, 1, 2, 3, 4a, 4b, 4c, 5, and 6. Lesions in Category 3 were considered benign, whereas those in categories 4a, 4b, 4c, and 5 were considered malignant. The suspicious sonographic features of malignancy were as follows: irregular shape, ill-defined margins (spiculated or angular), heterogeneity, microcalcification, posterior echo attenuation, length-to-width ratio >1, blood flow grades II–III, and abnormal axillary lymph nodes.

In the qualitative SWE analysis, lesions showing a maximal red color were referred to as stiff and those showing maximal blue color as soft. Quantitative SWE analysis was based on measurements performed on each SWE image and included the mean value of the entire lesion (SWEmean), maximum value (SWEmax, the ROI placed on the stiffest area), surrounding fat tissue (SWEfat, preferably at the same depth as the lesion), and lesion-to-fat velocity ratio (R, calculated using the acquired SWEmax and SWEfat). All five SWE images were analyzed, and the average values for SWEmean, SWEmax, and R were recorded.

CEUS analysis was based on our clinical experience and previous studies (19, 20). The following parameters were recorded: enhancement intensity (no, hypo-, iso-, or hyper-enhancement compared to the surrounding breast tissue), time (synchronous, earlier, or later enhancement compared to the surrounding breast tissue), direction (from the periphery inward, from the inside to the periphery, or all simultaneously), and pattern (presence or absence of ring and crab claw-like patters), and internal homogeneity, perfusion defect, contrast agent retention, and penetrating vessel. The following parameters were measured at the peak enhancement inside the lesion: enhanced area enlargement and the lesion’s size, margin, and shape. The CEUS BI-RADS scores were determined using the five-score system proposed by Xiao et al. (5). The following CEUS features were considered: enhancement homogeneity (heterogeneous, homogeneous), enhancement margin (not circumscribed, circumscribed), perfusion defect (present, absent), early hyperenhancement (present, absent), penetrating vessel (present, absent), and enhanced area enlargement (yes, no).

### Histopathological examination

All patients underwent breast coarse-needle biopsy or surgery within three days of performing the multi-parameter US examinations. Typical sections were processed and stained with hematoxylin and eosin for histopathology examinations. For patients who underwent both breast needle biopsy and surgery, the histopathology results after surgery were used as the final diagnosis of the lesions. The tissue sections were examined by experienced pathologists who were blinded to the clinical information. The histopathology results were considered the reference standard for the lesion.

### Statistical analysis

Statistical analysis was performed using IBM SPSS Statistics for Window, Version 26.0 (IBM Corp., Armonk, NY, USA). Quantitative data (i.e., patient age and lesion size) are expressed as mean ± standard deviation and were compared by the Student’s *t*-test. The chi-squared test compared categorical variables. Univariate and multivariable logistic regression analyses were used successively to determine predictors for malignancy using Cohort 1. Once the predictive model was established, the regression coefficient (β), standard error (SE), the results of the hypothesis test commonly used for the regression coefficient (Wald χ^2^), and odds ratios (ORs) with their 95% confidence intervals (CIs) were recorded. The diagnostic performance of the predictive model and conventional US BI-RADS were assessed by plotting receiver operating characteristic (ROC) curves and assessing the areas under them (AUC). Sensitivity and specificity were calculated using ROC analysis. The best cutoff values were obtained using the Youden index (maximum sensitivity + specificity – 1). *P* values < 0.05 were considered statistically significant.

## Results

### Lesion characteristics

The quantitative data for the included lesions in Cohorts 1, 2, and 3 are shown in **Table 1**. The malignancy rate in Cohorts 1, 2, and 3 was 39.1% (175/448), 40.3% (48/119), and 48.6% (52/107), respectively. The SWEmean value of the malignant lesions was significantly higher than the benign ones.

**Table 1:** Quantitative data for breast lesions in Cohorts 1, 2, and 3.

Characteristic	Cohort 1		Cohort 2		Cohort 3	
	Benign (*n* = 273)	Malignant (*n* = 175)	Benign (*n* = 71)	Malignant (*n* = 48)	Benign (*n* = 55)	Malignant (*n* = 52)
Mean age (years)	41.11 ± 11.86 (range, 18~88)	53.90 ± 13.38 (range, 23~94)	41.68 ± 12.25 (range, 22~80)	54.02 ± 13.90 (range, 22~85)	45.69 ± 14.83 (range, 22~84)	60.83 ± 12.30 (range, 37~87)
Age ≤ 40 years	145 (53.1%, 145/273)	27 (15.4%, 27/175)	35 (49.3%, 35/71)	7 (14.6%, 7/48)	21 (38.2%, 21/55)	3 (5.8%, 3/52)
Age > 40 years	128 (46.9%, 128/273)	148 (84.6%, 148/175)	36 (50.7%, 36/71)	41 (85.4%, 41/48)	34 (61.8%, 34/55)	49 (94.2%, 49/52)
Mean lesion size on US (mm)	16.57 ± 9.37 (range, 2.80~72.00)	19.43 ± 8.40 (range, 5.50~52.00)	19.44 ± 12.30 (range, 4.70~67.00)	20.94 ± 9.72 (range, 7.10~56.00)	15.04 ± 9.09 (range, 3.00~63.00)	20.42 ± 12.72 (range, 4.40~71.00)
Size ≤ 20 mm	201 (73.6%, 201/273)	113 (64.6%, 113/175)	47 (66.2%, 47/71)	26 (54.2%, 26/48)	45 (81.8%, 45/55)	33 (63.5%, 33/52)
Size > 20 mm	72 (26.4%, 72/273)	62 (35.4%, 62/175)	24 (33.8%, 24/71)	22 (45.8%, 22/48)	10 (18.2%, 10/55)	19 (36.5%, 19/52)
Mean lesion size on CEUS (mm)	17.10 ± 9.04 (range, 3.50~61.30)	24.87 ± 10.24 (range, 6.00~55.30)	20.15 ± 11.35 (range, 3.50~51.60)	25.33 ± 10.47 (range, 8.70~57.00)	15.89 ± 9.74 (range, 4.60~63.00)	27.12 ± 14.28 (range, 7.80~80.00)
Mean value of SWEmean (kPa)	20.91 ± 3.43 (range, 5.07~137.50)	59.41 ± 6.13 (range, 8.07~195.86)	23.86 ± 2.65 (range, 3.37~93.41)	46.57 ± 3.37 (range, 9.72~110.90)	16.43 ± 1.12 (range, 6.75~63.48)	38.02 ± 4.11 (range, 9.72~104.43)

US, ultrasonography; CEUS, contrast-enhanced ultrasonography; SWEmean, mean shear wave elastography.

### Predictive model development

Univariate analyses showed that the length-to-width ratio > 1 on conventional US and perfusion defects and penetrating vessels on CEUS could not predict breast tumor malignancy (all *P* > 0.05). Multivariable analysis showed the following independent predictors for diagnosing breast tumor malignancy (**Table 2**): age over 40 years (OR, 5.337; *P* < 0.001), ill-defined margin on conventional US (OR, 4.844; *P* = 0.008), heterogeneity on conventional US (OR, 2.171; *P* = 0.016), rich blood flow on conventional US (OR, 3.335; *P* < 0.001), abnormal axillary lymph nodes on conventional US (OR, 9.174; *P* = 0.004), enhanced area enlargement on CEUS (OR, 2.836; *P* = 0.001), contrast agent retention on CEUS (OR, 4.800; *P* = 0.008), irregular shape on CEUS (OR, 3.828; *P* < 0.001), and SWEmean > cutoff value (OR, 6.295; *P* < 0.001).

**Table 2:** Results of the multivariable analysis for breast cancer prediction using multi-parameter ultrasonography.

Indicator	β	SE	Wald χ^2^	*P*-value	OR	95% CI of OR
Age > 40 years	1.675	0.356	22.176	<0.001	5.337	[2.658, 10.716]
Ill-defined margin on US	1.578	0.590	7.139	0.008	4.844	[1.523, 15.408]
Heterogeneity on US	0.775	0.323	5.764	0.016	2.171	[1.153, 4.087]
Rich blood flow on US	1.205	0.343	12.364	<0.001	3.335	[1.704, 6.527]
Abnormal axillary lymph nodes on US	2.216	0.772	8.237	0.004	9.174	[2.019, 41.679]
Enhanced area enlargement on CEUS	1.042	0.323	10.433	0.001	2.836	[1.507, 5.339]
Contrast agent retention on CEUS	1.569	0.587	7.145	0.008	4.800	[1.520, 15.164]
Irregular shape on CEUS	1.342	0.348	14.899	<0.001	3.828	[1.936, 7.569]
SWEmean > cutoff value	1.840	0.317	33.724	<0.001	6.295	[3.383, 11.712]
Constant	-20.360	2.468	68.071	<0.001	<0.001	

SE, standard error; OR, odds ratio; CI, confidence interval; US, ultrasonography; CEUS, contrast-enhanced ultrasonography; SWEmean, mean shear wave elastography.

Based on the data in **Table 2**, we established the following logistic model:

*p* = 1/1 + Exp ∑ [–20.360 + 1.675 × (if age > 40 y) + 1.578 × (if spiculated margin on US) + 0.775 × (if heterogeneous on US) + 1.205 × (if rich blood flow on US) + 2.216 × (if abnormal axillary lymph nodes on US) + 1.042 × (if enhanced area enlargement on CEUS) + 1.569 × (if contrast agent retention on CEUS) + 1.342 × (if irregular shape on CEUS) + 1.840 × (if SWEmean > cutoff value)].

If one of the indexes is positive, it will be defined as 1. Otherwise, it will be defined as 0. The final result *p* greater than 0 indicates a benign lesion, while *p* less than 0 indicates a malignant lesion.

### Validation and evaluation of the predictive model performance

**Figure 2:**
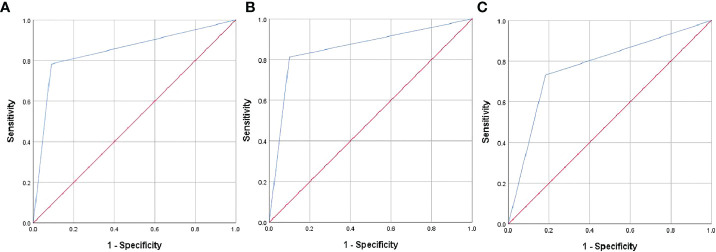
Receiver operating characteristic (ROC) curves of the predictive model in Cohorts 1 **(A)**, 2 **(B)**, and 3 **(C)**.

**Figure 3:**
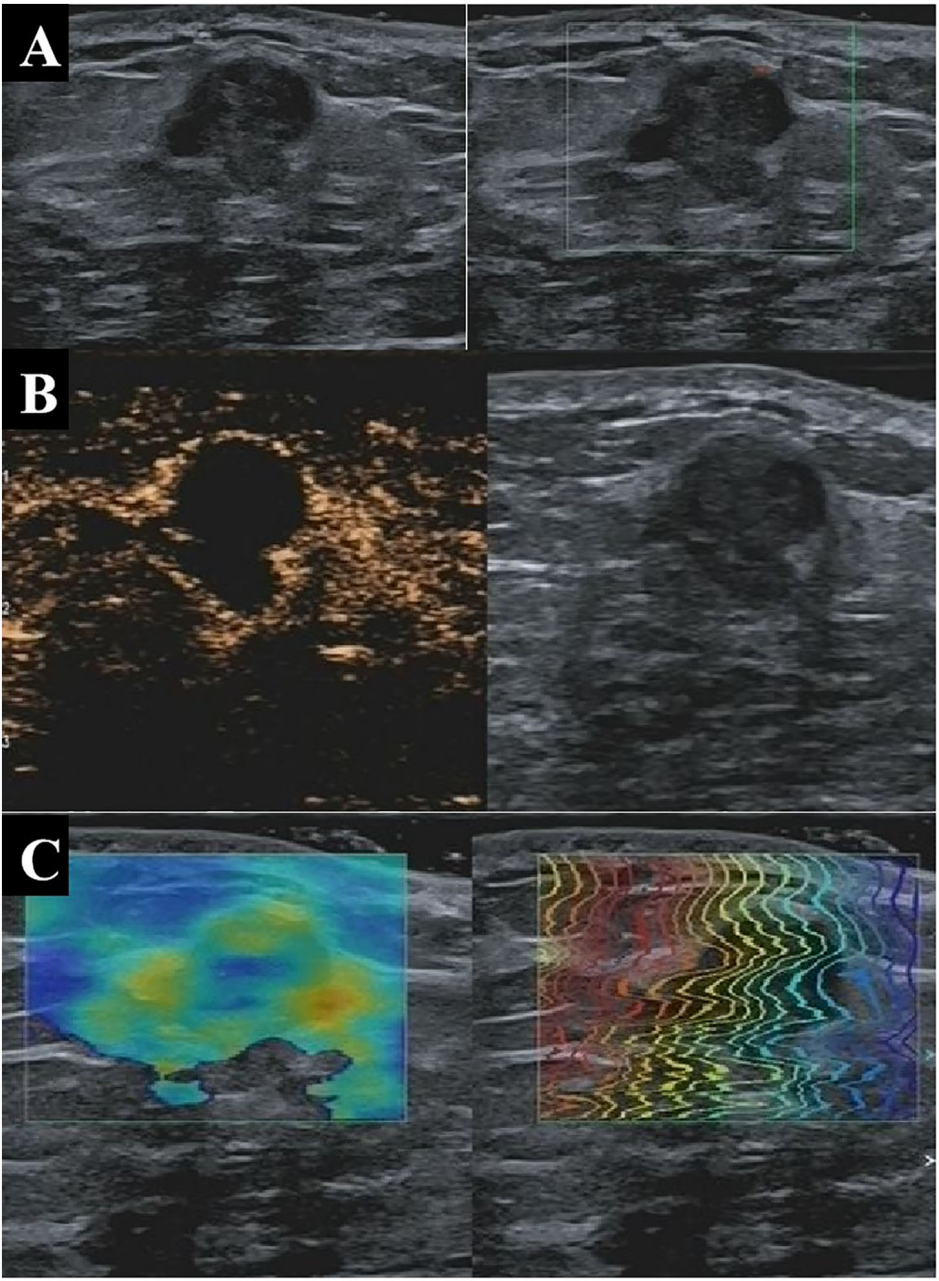
A 33-year-old patient with pathologically confirmed breast adenosis showing a hypoechoic solid nodule at 4 o’clock in the right breast with well-defined margins, irregular shape, and poor blood flow on conventional US **(A)**, indicating a malignant lesion with a BI-RADS 4b score. On CEUS, the tumor appears as a nodule without blood perfusion **(B)**, indicating a benign lesion with a BI-RADS 3 score. The nodule was soft based on shear wave elastography, indicating that the lesion was benign **(C)**. The nodule was assessed as benign by the multi-parameter predictive model. US, ultrasonography; CEUS, contrast-enhanced ultrasonography; BI-RADS, Breast Imaging Reporting and Data System.

**Figure 4:**
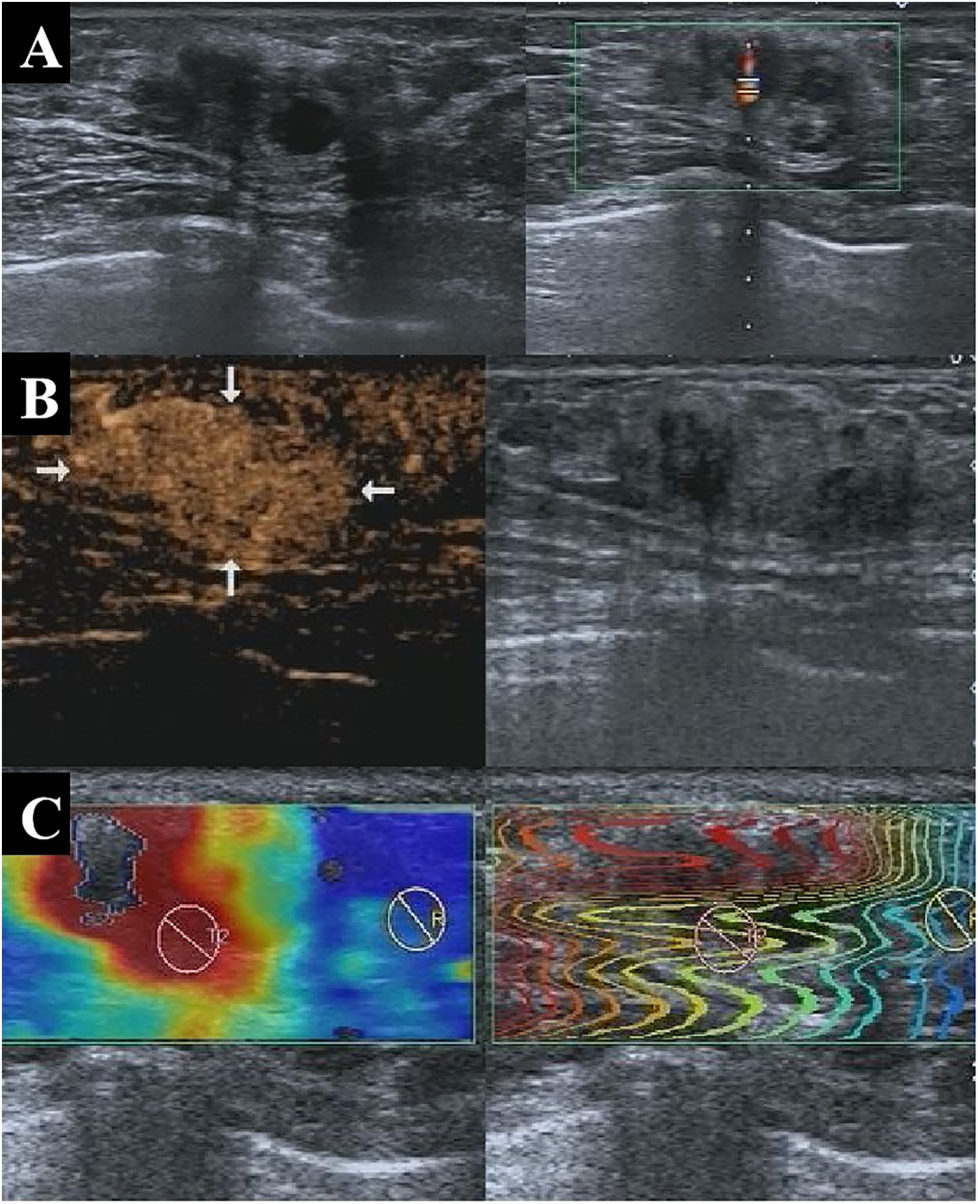
A 47-year-old patient with pathologically confirmed invasive ductal carcinoma showing an isoechoic solid nodule at 1 o’clock in the left breast with well-defined margins and a regular shape on conventional US **(A)**, indicating a benign lesion with a BI-RADS 3 score. CEUS showed early hyperenhancement, enhanced area enlargement, contrast agent retention, clear margins, and irregular shape **(B)**, indicating a malignant lesion with a BI-RADS 4b score. SWE also indicated that the lesion was malignant **(C)**. The nodule was assessed as malignant by the multi-parameter predictive model. US, ultrasonography; CEUS, contrast-enhanced ultrasonography; BI-RADS, Breast Imaging Reporting and Data System; SWE, shear wave elastography.

The model’s performance was analyzed in terms of accuracy, sensitivity, specificity, and AUC. The respective values for Cohort 2 were 86.6%, 90.1%, 81.3%, and 0.857. The AUC values for Cohorts 1 and 3 were 0.847 and 0.774, respectively (**Figures 2A–C**), indicating that the model had a favorable diagnostic value. This was further confirmed in two cases (**Figures 3** and **4**) in which the conventional US was wrong while the model was correct.

When using data of Cohort 1, the predictive model showed significantly higher sensitivity (91.2% vs. 60.0%), specificity (78.3% vs. 34.8%), and accuracy (86.2% vs. 58.7%) than conventional US. When compared to conventional US, the model showed significantly higher specificity (81.3% vs. 31.0% and 73.1% vs. 7.3%) and accuracy (86.6% vs. 58.8% and 77.6% vs. 52.3%) in Cohorts 2 and 3, respectively, without a significant loss of sensitivity (from 100.0% to 90.1% in Cohort 2 and from 100.0% to 81.8% in Cohort 3; **Table 3**).

**Table 3:** Comparison of the diagnostic performance between conventional US and the predictive model in Cohorts 1, 2, and 3.

Parameter	Cohort 1		Cohort 2		Cohort 3	
	US	Model	US	Model	US	Model
Specificity	34.8%	78.3%	31.0%	81.3%	7.3%	73.1%
Sensitivity	60.0%	91.2%	100.0%	90.1%	100.0%	81.8%
Accuracy	58.7%	86.2%	58.8%	86.6%	52.3%	77.6%
AUC	0.654	0.847	0.655	0.857	0.536	0.774

US, ultrasonography; AUC, area under the receiver operating characteristic curve.

### Reduction of unnecessary biopsies by the predictive model

Cases of unnecessary biopsies were those with BI-RADS > 3 based on conventional US but pathologically proven benign. The number of lesions with conventional US BI-RADS > 3 in Cohort 1 that were correctly downgraded by the predictive model was 73 (of 109 downgraded lesions), indicating an unnecessary biopsy reduction rate of 67.0% (73/109). Similarly, the unnecessary biopsy reduction rate in Cohort 2 was 75.0% (27/36) and 70.8% (34/48) in Cohort 3.

## Discussion

Conventional US, SWE, and CEUS play distinct and important roles in breast cancer diagnosis. However, the diagnostic value of each single approach is insufficient to accurately diagnose malignant breast lesions. Multi-parameter US can assess the morphology, elasticity, and blood supply of these lesions. This study aimed to establish, validate, and evaluate a predictive model based on multi-parameter US in clinical breast cancer diagnosis, exploring whether it can improve the diagnostic efficiency and reduce unnecessary breast biopsies. We first built a model analyzing data of 448 patients by univariate and multivariable analyses. Subsequently, we validated the model in Cohort 2 (119 patients) and evaluated it in Cohort 3 (107 patients). The model’s diagnostic value was compared to conventional US. The results of Cohorts 2 and 3 showed that the model had a great potential for use in clinical practice.

As shown in **Table 2**, nine indicators were included in the model: age over 40 years; conventional US findings, including ill-defined margin, tumor heterogeneity, rich blood flow, and abnormal axillary lymph nodes; CEUS findings, including enhanced area enlargement, contrast agent retention, and irregular shape; SWEmean larger than the cutoff value. Patients with malignant lesions were shown to be older than those with benign lesions (17, 21), consistent with our finding. Ill-defined margins can be attributed to the invasion of malignant tumors into the surrounding breast tissue (22–24), confirmed by our findings. Similarly, Zhang et al. (25) reported that the appearance of heterogeneity on US images indicated faster proliferation and worse prognosis, consistent with our findings. Our study also showed similar findings related to disordered and disseminated blood flow. Rich blood flow is an indicator of the faster growth and higher metabolism of malignant tumors over benign tumors (26, 27). Abnormal lymph nodes on US images, a possible indicator of high tumor aggressiveness, could also help detect breast cancers, as previously reported (28, 29).

Among the findings indicating malignancy on CEUS images, enhanced area enlargement is widely recognized (17, 30). Although the area measured by CEUS for malignant lesions was reportedly closer to the pathological findings than conventional US (31), the measurements included the breast lesions and blood-rich areas around them. As normal tissue blood flow cannot maintain tumor growth, the surrounding tissues stimulate angiogenesis to support tumor growth (32). Contrast agent retention revealed that the disordered blood vessel distribution inside the tumor led to poor venous return. The irregular shape shown on CEUS images was also found in other studies (30, 33), and attributed to the abundant and disorganized blood flow in malignant tumors and their infiltration into surrounding breast tissue. The SWE value refers to the lesion’s stiffness. Malignant lesions tend to be stiffer than benign lesions and show higher SWE values, as previously confirmed (7, 8, 34).

The diagnostic performance of the predictive model in Cohorts 1, 2, and 3 was greater than by conventional US. Previous studies also compared multi-parameter US models to conventional US and achieved similar results (16–18). The sensitivity of the conventional US was 100.0% in Cohorts 2 and 3, but the specificity was rather low (31.0% and 7.3%, respectively). Low specificity might lead to many unnecessary biopsies. Therefore, it is important to maintain a balance between sensitivity and specificity in clinical practice. After combining data from conventional US, CEUS, and SWE, the specificity of the predictive model significantly improved to 81.3% and 73.1% in Cohorts 2 and 3, respectively, without a significant loss in sensitivity. These improvements could greatly help clinical practice by reducing unnecessary biopsies, as shown in the fourth part of results.

Some previous studies have highlighted the valuable diagnostic performance of multi-parameter US in breast cancer. For example, the study by Li et al. (17) developed, validated, and evaluated a prediction model for malignancy in BI-RADS 4 breast lesions, while our study evaluated all breast lesion types. Moreover, our study included quantitative analysis of SWE, while their study included only qualitative analysis. Still, both studies achieved equivalent diagnostic performances based on multi-parameter US.

This study had several limitations. First, the malignancy rate was relatively high, possibly because most benign lesions were followed up. This may have led to some mistakes. Second, this was a retrospective, single-center study, so the number of the cases was limited. A larger number of patients from multiple centers is needed to confirm our results. Finally, this study did not assess the repeatability of quantitative parameters such as the SWE measurements, which should be explored in future studies.

## Conclusion

The multi-parameter US model showed good performance in diagnosing breast cancer. The model established in this study could improve the diagnostic specificity without a significant loss in sensitivity, helping reduce unnecessary biopsies and guide clinical diagnosis and treatment.

## Data availability statement

The original contributions presented in the study are included in the article/supplementary material. Further inquiries can be directed to the corresponding authors.

## Ethics statement

The studies involving human participants were reviewed and approved by Shanghai General Hospital, Shanghai Jiao Tong University School of Medicine. The patients/participants provided their written informed consent to participate in this study. Written informed consent was obtained from the individual(s) for the publication of any potentially identifiable images or data included in this article.

## Author contributions

Guarantor of integrity of the entire study: RW, MY; Study concepts and design: JC, RW, MY; Literature research: JC, JM, SS, YS; Clinical studies: JC, JM, CL, SS, YS; Experimental studies/data analysis: JC, JM, CL, MY; Statistical analysis: JC, SS, MY; Manuscript preparation: JC, JM; Manuscript editing: JC, JM, RW, MY. All authors contributed to the article and approved the submitted version.

## Funding

This work was supported by the National Natural Science Foundation of China (Grants No. 82071931, 82130057, 82171951), Shanghai Outstanding Young Medical Talents Training Plan (2022YQ044), the interdisciplinary program of Shanghai Jiaotong University (ZH2018ZDA17), the program from Science and Technology Commission of Shanghai Municipality (No. 20Y11912400), and the 2019 clinical research innovation team of Shanghai General Hospital (No. CTCCR-2019B05).

## Conflict of interest

The authors declare that the research was conducted in the absence of any commercial or financial relationships that could be construed as a potential conflict of interest.

## Publisher’s note

All claims expressed in this article are solely those of the authors and do not necessarily represent those of their affiliated organizations, or those of the publisher, the editors and the reviewers. Any product that may be evaluated in this article, or claim that may be made by its manufacturer, is not guaranteed or endorsed by the publisher.

## Abbreviations

AUC, area under the receiver operating characteristic curve; BI-RADS, Breast Imaging Reporting and Data System; CEUS, contrast-enhanced ultrasonography; CI, confidence interval; OR, odds ratio; ROC, receiver operating characteristic; ROI, region of interest; SE, standard error; SWE, shear wave elastography; US, ultrasonography
